# Efficacy and safety of widely used treatments for macular oedema secondary to retinal vein occlusion: a systematic review

**DOI:** 10.1186/1471-2415-14-7

**Published:** 2014-01-21

**Authors:** Julie Glanville, Jacoby Patterson, Rachael McCool, Alberto Ferreira, Kerry Gairy, Ian Pearce

**Affiliations:** 1 York Health Economics Consortium, University of York, York, UK; 2 Novartis, Basel, Switzerland; 3 Novartis, Frimley, UK; 4 St Paul’s Eye Unit, Royal Liverpool University Hospitals NHS Trust, Liverpool, UK

**Keywords:** Retinal vein occlusion, Ranibizumab, Dexamethasone intravitreal, Laser, Branch retinal vein occlusion, Central retinal vein occlusion

## Abstract

**Background:**

Macular oedema secondary to retinal vein occlusion (RVO) can cause vision loss due to blockage of the central retinal vein (CRVO) or a branch retinal vein (BRVO). This systematic review assessed the efficacies of widely used treatments for macular oedema secondary to RVO and the feasibility of conducting indirect comparisons between these therapies.

**Methods:**

A systematic review was undertaken in November 2010, including a literature search for trials in medical databases and relevant websites. Abstracts, conference presentations and unpublished studies were considered. Studies were data-extracted and quality assessed by two independent researchers. Outcome measures included the mean change in best corrected visual acuity (BCVA) from baseline in the study eye and/or number of patients gaining at least 10 letters from baseline to 6 months or the nearest equivalent time point.

**Results:**

Fourteen unique randomized controlled trials (RCTs) were identified. Ranibizumab 0.5 mg produced greater improvements in BCVA at 6 months than sham in BRVO (mean difference 11.0 letters, 95% confidence interval [CI] 7.83, 14.17) and CRVO (mean difference 14.10 letters, 95% CI 10.51, 17.69) in two double-blind sham-controlled RCTs. Pooled data from two double-blind, sham-controlled RCTs showed that improvements in BCVA were also significantly better for dexamethasone intravitreal (IVT) implant 0.7 mg compared with sham in patients with BRVO or CRVO (mean difference 2.5 letters, 95% CI 0.7, 4.3); the difference was significant for BRVO alone, but not CRVO alone. A significantly greater proportion of patients with BRVO gained ≥15 letters with laser therapy vs. no treatment at 36 months in a large prospective RCT (odds ratio 3.16, 95% CI 1.25, 8.00), whereas no difference was observed at 9 months in a smaller study. Three studies reported no benefit for laser therapy in CRVO. No indirect comparisons with ranibizumab were feasible due to differences in study design and baseline characteristics.

**Conclusions:**

Data from RCTs for ranibizumab and dexamethasone IVT demonstrate that both new agents constitute significant improvements over the previously widely accepted standard of care (laser therapy) for the treatment of BRVO and CRVO. However, head-to-head studies are needed to assess the relative efficacies of licensed therapies for RVO.

## Background

Retinal vein occlusion (RVO) is the second most common retinal vascular disease after diabetic retinopathy and is an important cause of vision loss [1]. It is caused by occlusion of veins at the back of the eye, which become occluded by vascular clot, external compression or vessel wall pathology [2]. Occlusion can occur either in the central retinal vein (central RVO, CRVO) or branches of the retinal veins (branch RVO, BRVO) that combine to form the central vein; prognoses and outcomes vary depending on which is occluded [3]. RVO can lead to fluid leakage from capillaries draining into the obstructed vein, caused in part by secretion of vascular endothelial growth factor (VEGF) and interleukin-6, and resulting in thickening of the retina (oedema) [4]. Macular oedema is the most common cause of vision loss from RVO [5]. If left untreated, patients with BRVO will gain on average only 0.23 lines on the Early Treatment of Diabetic Retinopathy Study (ETDRS) scale after 3 years, to an average level of 20/70, but full recovery of vision is generally not achieved owing to persistent oedema and resulting structural damage [6]. Prognosis is worse for patients with macular oedema secondary to CRVO, with visual acuity (VA) declining over time if left untreated [7]. Both BRVO and CRVO are associated with significant impairments in vision-related quality of life (as measured by the National Eye Institute visual function questionnaire, NEI-VFQ) [8,9].

A number of therapies are currently available for the treatment of RVO. Laser photocoagulation has been the standard of care for treatment of BRVO in the UK [4] based on the results of the BVOS study performed 30 years ago [6]. However, poor vision persists despite photocoagulation treatment in many patients, and its use is not recommended until 3 months after development of BRVO [4]. Laser therapy was also investigated in patients with CRVO, but was found to produce no improvement in VA over no treatment [10]; hence, observation was the standard of care for CRVO in the UK for several decades [4]. Two new treatments – ranibizumab (Lucentis®, Novartis AG, Basel, Switzerland) and dexamethasone intravitreal (IVT) implant (Ozurdex®, Allergan, Irvine, CA, USA) – have recently been approved for treatment of macular oedema secondary to RVO in the UK, Europe and USA [11-14]. Ranibizumab is a recombinant, humanized, monoclonal antibody fragment developed specifically for IVT use, which binds with high affinity to multiple VEGF isoforms and prevents binding of VEGF to VEGF receptors 1 and 2 [15]; it is prescribed at a dose of 0.5 mg. Dexamethasone IVT is a sustained-biodegradable implant containing the corticosteroid dexamethasone. Corticosteroids including dexamethasone are known to have anti-inflammatory, anti-angiogenic properties and may inhibit the expression of VEGF and other proinflammatory cytokines such as IL-6, ICAM-1 and MCP-1 [12,16-22]; it is prescribed at a dose of 0.7 mg. Two further therapies are also used to treat RVO. Bevacizumab, a full-length anti-VEGF antibody developed for treatment of cancer, has not been developed or licensed for IVT use; however, it is sometimes used to treat RVO. IVT triamcinolone (IVTA), a corticosteroid injection, with a similar mechanism of action to dexamethasone [16,23,24], is used off-label for the treatment of RVO. Since this review was undertaken, a third anti-VEGF treatment – aflibercept (Eylea®, Bayer AG, Berlin, Germany) – has been approved for treatment of macular oedema secondary to RVO [25].

It is important to consider the relative efficacy of the available therapies for RVO with published data. This systematic review was therefore performed to assess the efficacy and safety of available treatments for RVO as reported in randomized controlled trials (RCTs), and to assess the feasibility of conducting indirect comparisons between ranibizumab and other therapies available in the UK.

## Methods

This systematic review was conducted using systematic review methodology based on the Centre for Reviews and Dissemination’s Guidance for Undertaking Systematic Reviews [26]. The systematic review was conducted according to a written protocol that defined the research question, the inclusion criteria and methods for study selection, criteria for assessment of study quality, the data to be extracted and the analyses to be performed.

### Searches and data extraction

A systematic literature search was performed on 18 November 2010 in core medical databases (Medline, Embase, the Cochrane Library, Cumulative Index to Nursing and Allied Health Literature), and further searches were performed in relevant websites including the International Clinical Trials Registry Platform and the Association of Research and Vision and Ophthalmology. The main interventions included in the searches were ranibizumab, bevacizumab, dexamethasone IVT and laser photocoagulation. Data for other interventions were included only for comparisons with the main interventions. Details of the search used for Medline are included in Additional file 1 and study inclusion criteria are summarized in Table 1. Abstracts, conference presentations and unpublished studies were considered eligible for inclusion in the review if they met the inclusion criteria; only RCTs in English were included. Additional sources of data included clinical study reports for ranibizumab [27-31], the dexamethasone IVT manufacturer’s submission to the National Institute for Health and Clinical Excellence (NICE) for dexamethasone IVT and the Evidence Review Group response [32,33] and results of the Global EvaluatioN of implantable dExamethasone in retinal Vein occlusion with macular edemA (GENEVA) studies reported within the ClinicalTrials.gov records [34,35].

**Table 1:** Inclusion criteria according to the population–intervention–comparison–outcome (study design) model

**Population**	**Patients with clinically significant BRVO or CRVO**
Intervention	1. Ranibizumab
	2. Bevacizumab
	3. Dexamethasone IVT
	4. Laser photocoagulation
Comparison	Any of the interventions listed above and any of the following:
	1. Best supportive care
	2. Grid pattern photocoagulation
	3. Sham injections
	4. Mixed treatment comparisons
Outcomes	Data for BRVO and CRVO were extracted separately where possible
	*Primary measures (at least one of the following extracted for every study)*
	1. Mean change in BCVA from baseline
	2. Number of patients gaining ≥ 10 letters from baseline to 6 months
	*Secondary measures extracted if available*
	1. Number of patients gaining ≥ 15 letters
	2. AEs
	3. SAEs
	4. Vision-related quality of life
Study design	Randomized controlled trials of any publication date

*AE*, adverse event; *BCVA*, Best corrected visual acuity; *BRVO*, Branch retinal vein occlusion; *CRVO*, Central retinal vein occlusion; *IVT*, Intravitreal; *SAE*, serious adverse event.

The screening process was carried out by a single researcher and checked by a second researcher; discrepancies were resolved through discussion or a third researcher (JG, FC, SB). Data extraction was carried out independently by two researchers and discrepancies were resolved by discussion (JG, JP, FC, SB).

### Outcome measures

When possible, data for BRVO and CRVO were extracted separately. Mean change in best corrected VA (BCVA) from baseline in the study eye and/or the number of patients gaining at least 10 letters from baseline to 6 months, or nearest equivalent time point, were extracted for all studies (Tables 2 and 3); data for the number of patients gaining at least 15 letters were also extracted if available. These outcome measures were chosen because a gain in BCVA of at least 10 letters has been shown to be associated with a clinically relevant improvement in vision-related quality of life [36-38], and a gain of at least 15 letters is recommended by the US Food and Drug Administration as the primary endpoint measure for treatments for visual impairment [39]. Other outcomes extracted if available included adverse events (AEs), serious AEs (SAEs) and vision-related quality of life.

**Table 2:** Study design and key efficacy data for RCTs investigating treatments for BRVO (efficacy data are presented at 6 months unless otherwise indicated)

**Study design and patient characteristics**	**BRAVO [**[43]**]**	**Pooled data from both GENEVA trials [**[18]**]**	**Battaglia Parodi et al [**[45]**] (data presented at 9 months)**	**BVOS group [**[6]**] (data presented at 36 months)**	**Russo et al [**[50]**]**	**Moradian et al [**[49]**] (data presented at 6 weeks)**
Study design	Blinded RCT	Blinded RCT	RCT (blinding not reported)	Blinded RCT	Unblinded RCT	Blinded RCT
Study quality^a^	6/8	7/8	3/8	7/8	5/8	7/8
Treatment arms	1. RBZ 0.3 mg	1. Dex IVT 0.7 mg	1. Laser	1. Laser	1. Laser	1. IVB
	2. RBZ 0.5 mg	2. Dex IVT 0.35 mg	2. No treatment	2. No treatment	2. IVB	2. Sham
	3. Sham (laser)	3. Sham				
Key inclusion/exclusion criteria	Age ≥ 18 years	Age ≥ 18 years	VA < 0.6	VA ≤ 20/40	logMAR ETDRS ≤ 0.4	BCVA ≤ 20/50
	ETDRS BCVA: 20/50–20/400	BCVA < 20/50			CMT ≥ 30 μm	
	Mean CST ≥ 250 μm					
No. eyes (patients) randomized per arm	1. 134	1. 291	1. 33	1. 43	1. 15	1. 42
	2. 131	2. 260	2. 35	2. 35	2. 15	2. 39
	3. 132	3. 279				
Study duration	6 months	6 months	24 months	36 months	12 months	3 months
Efficacy						
Mean change in BCVA, mean (SD)	1. 16.6 (11.0)*	1. 7.4^b^*	1. 0.7 (0.2)	NR	1. 0.68 (0.13)	1. 0.49 (0.32)*
	2. 18.3 (13.2)*	2. NR	2. 0.7 (0.2)		2. 0.57 (0.16)	2. 0.75 (0.48)
	3. 7.3 (13.0)	3. 4.9^b^			logMAR	
Number of patients gaining	1. 74 (55.2)*	1. 67 (23.0)	NR	NR	1. 7 (46.7)	NR
≥ 15 letters (%)	2. 80 (61.1)*	2. NR			2. 11 (73.3)	
	3. 38 (28.8)	3. 56 (20.1)				
Number of patients gaining	1. 99 (73.9)*	1. 120 (41.2)*	NR	1. 28 (65.1)*	NR	NR
≥ 10 letters (%)	2. 103 (78.6)*	2. 55 (21.2)		2. 13 (37.1)		
	3. 53 (40.2)	3. 92 (33.0)				

^a^Study quality was judged on the following criteria: randomization, allocation, blinding, similarity of groups, loss to follow-up, imbalance between groups, reporting of data from intention-to-treat group and whether the study was free of selective reporting. Detailed assessment of study quality is presented in Additional file 2.

^b^Data taken from manufacturer’s submission to NICE [32] (standard deviations were not reported).

*Statistically significant compared with sham/no treatment.

*BCVA*, Best-corrected visual acuity; *BRAVO*, Ranibizumab for the Treatment of Macular Edema after BRAnch retinal Vein Occlusion: Evaluation of Efficacy and Safety; *BRVO*, Branch retinal vein occlusion; *BVOS*, Branch Retinal Vein Occlusion Study; *CMT*, Central macular thickness; *CST*, Central subfield thickness; *Dex IVT*, Dexamethasone intravitreal; *ETDRS*, Early Treatment Diabetic Retinopathy Study; *GENEVA*, Global EvaluatioN of implantable dExamethasone in retinal Vein occlusion with macular edemA; *IVB*, Intravitreal bevacizumab; *logMAR*, Logarithm of minimum angle of resolution; *RBZ*, Ranibizumab; *RCT*, Randomized controlled trial; *VA*, Visual acuity; *NR*, Not reported; *SD*, standard deviation.

**Table 3:** Study design and key efficacy data for RCTs investigating CRVO or CRVO and BRVO (efficacy data are presented at 6 months unless otherwise indicated)

**Study design and patient characteristics**	**CRUISE [**[42]**]**	**ROCC [**[44]**]**	**Pooled data from both GENEVA trials [**[18]**]**	**CVOS group [**[10]**] (data presented at 12 months)**	**Laatikainen et al [**[46]**] (data presented at 12 months)**	**May et al [**[47]**] (data presented at 24 months)**	**Faghihi et al [**[48]**]**	**Kuppermann et al [**[20]**] (BRVO and CRVO – data presented at 3 months)**
Study design	Blinded RCT	Blinded RCT	Blinded RCT	Blinded RCT	RCT (blinding not reported)	RCT (blinding not reported)	Blinded RCT	Blinded RCT
Study quality^a^	6/8	4/8	7/8	5/8	5/8	5/8	Poster only	7/8
Treatment arms	1. RBZ 0.3 mg	1. RBZ 0.5 mg	1. Dex IVT 0.7 mg	1. Laser	1. Laser	1. Laser	1. IVB	1. Dex IVT
	2. RBZ 0.5 mg	2. Sham	2. Dex IVT 0.35 mg	2. No treatment	2. No treatment	2. No treatment	2. Sham	0.7 mg/
	3. Sham (laser)		3. Sham					0.35 mg
								2. No treatment
Key inclusion/exclusion criteria	Age ≥ 18 years ETDRS: 20/50–20/320 Mean CST: ≥ 250 μm	Age ≥ 50 years ETDRS: 6–73 letters	Age ≥ 18 years BCVA < 20/50	VA 20/50–5/200	VA ≤ 6/24	Age > 40 years VA < 20/40	BCVA ≤ 20/50	Persistent macular oedema following laser
No. eyes (patients) randomized per arm	1. 132	1. 15	1. 136	1. 68	1. 24	1. 15	NR	1. 35
	2. 130	2. 14	2. 154	2. 72	2. 24	2. 19		2. 34
	3. 130		3. 147					
Study duration	6 months	6 months	6 months	36 months	12 months	28.5 months	NR	6 months
Efficacy								
Mean change in BCVA, mean (SD)	1. 12.7 (15.9)*	1. 12 (20.0)*	1. 0.1^b^	NR	NR	NR	NR	NR
	2. 14.9 (13.2)*	2. 1 (17.0)	2. NR					
	3. 0.8 (16.2)		3. −1.8^b^					
Number of patients gaining ≥ 15 letters (%)	1. 61 (46.2)*	NR	1. 24 (17.6)	NR	NR	NR	NR	NR
	2. 62 (47.7)*		2. 26 (16.9)					
	3. 22 (16.9)		3. 18 (12.2)					
Number of patients gaining ≥ 10 letters (%)	1. 82 (62.1)*	NR	1. 36 (26.5)	1. 10 (14.7)	1. 2 (8.3)	1. 3 (20.0)	NR	1. 31 (88.6)*
	2. 92 (70.8)*		2. NR	2. 6 (8.3)	2. 2 (8.3)	2. 5 (26.3)		2. 15 (44.1)
	3. 33 (25.4)		3. 35 (23.8)					

^a^Study quality was judged on the following criteria: randomization, allocation, blinding, similarity of groups, loss to follow-up, imbalance between groups, reporting of data from intention-to-treat group and whether the study was free of selective reporting. Detailed assessment of study quality is presented in Additional file 2.

^b^Data taken from manufacturer’s submission to NICE [32] (standard deviations were not reported).

*Statistically significant compared with sham/no treatment.

*BCVA*, Best-corrected visual acuity; *BRVO*, Branch retinal vein occlusion; *CMT*, Central macular thickness; *CRUISE*, Ranibizumab for the Treatment of Macular Edema after Central Retinal Vein OcclUsIon Study: Evaluation of Efficacy and Safety; *CVOS*, Central Retinal Vein Occlusion Study; *CRVO*, Central retinal vein occlusion; *CST*, Central subfield thickness; *Dex IVT*, Dexamethasone intravitreal; *ETDRS*, Early Treatment Diabetic Retinopathy Study; *GENEVA*, Global EvaluatioN of implantable dExamethasone in retinal Vein occlusion with macular edemA; *IVB*, intravitreal bevacizumab; *RBZ*, Ranibizumab; *RCT*, randomized controlled trial; *ROCC*, Study Comparing Ranibizumab to Sham in Patients with Macular Edema Secondary to Central Retinal vein OCClusion; *VA*, Visual acuity; *NR*, Not reported; *SD*, standard deviation.

### Quality assessment, risk of bias and feasibility of performing indirect comparisons

The quality and potential risk of bias of included studies were assessed according to the minimum criteria specified by the NICE guidelines [40]. Key points assessed included method of randomization, blinding protocols and baseline patient demographics (full details in Additional file 2). Potential sources of bias were participants or care providers not blinded to treatment and unexpected imbalances in rates of drop-out between groups. Evidence to suggest that other outcome measures were also assessed was recorded. The feasibility of performing indirect comparisons was assessed according to guidance from the Australian Pharmaceutical Benefits Advisory Board, because these are the only well established guidelines currently available [41].

### Statistical analysis

For dichotomous outcomes, results were presented as Mantel–Haenszel odds ratios (ORs), with 95% confidence intervals (CIs). For continuous outcomes, results were presented as mean differences, with 95% CIs if data on mean and standard deviation were identifiable, and pooled data were analysed using weighted mean differences. Indirect comparisons were to be performed dependent upon the suitability of the data.

## Results

**Figure 1:**
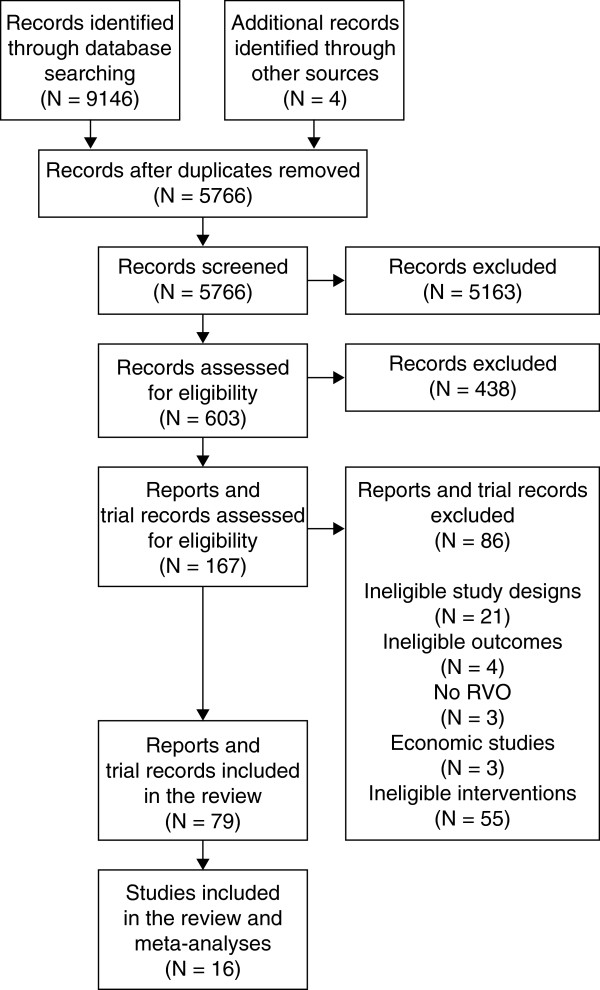
**Screening and selection of relevant references.** RVO, retinal vein occlusion.

In total, 5766 unique references were identified from the searches. From these, 14 studies met the inclusion criteria. The screening and selection process is shown in Figure 1.

### Studies

Fourteen unique RCTs were included in the review. Three studies compared ranibizumab with sham injection [42-44], three studies compared dexamethasone IVT with sham injection [18,20], five studies compared laser therapy with no treatment [6,10,45-47] and bevacizumab was compared with sham injections in two studies [48,49] and with laser therapy in another study [50]. Efficacy data for mean change in BCVA and the percentage of patients gaining at least 10 or at least 15 letters and key study details are provided in Tables 2 and 3. Key comparative efficacy data are summarized in Table 4.

**Table 4:** Efficacy comparator analysis for BRVO and CRVO (all data are presented at 6 months unless otherwise stated)

	**0.3 mg RBZ vs. sham**	**0.5 mg RBZ vs. sham**	**Dex IVT 0.35 mg vs. sham**	**Dex IVT 0.7 mg vs. sham**	**Laser vs. no treatment/observation**	**IVB vs. sham**	**IVB vs. laser**
*BRVO*							
Mean change in BCVA (measured by ETDRS scale unless otherwise specified), mean difference (95% CI)	9.30* (6.40, 12.20) [43]	11.0* (7.83, 14.17) [43]	NR	2.5* (0.6, 4.3) [18]	Battaglia Parodi et al (9 months), -0.01, (-0.08, +0.06)^a^[45]	6 weeks, logMAR; –0.26* (–0.44, –0.08)^b^[49]	logMAR; –0.11 (–0.01, –0.21)^b^[50]
Number of patients gaining ≥ 15 letters, OR (95% CI)	3.05* (1.84, 5.07) [43]	3.88* (2.32, 6.49) [43]	NR	1.19 (0.80, 1.78) [18]	NR	NR	logMAR; 3.14 (0.68, 14.5) [50]
Number of patients gaining ≥ 10 letters, OR (95% CI)	4.22* (2.51, 7.09) [43]	5.48* (3.18, 9.44) [43]	1.07 (0.70, 1.62) [18]	1.43* (1.01, 2.01) [18]	BVOS (36 months), [6] 3.16* (1.25, 8.00)	NR	NR
*CRVO*							
Mean change in BCVA, mean difference (95% CI)	11.9* (8.01, 15.79) [42]	CRUISE, 14.10* (10.51, 17.69) [42] ROCC, 11.0* (–2.48, 24.48) [44]	NR	NR, NS^c^	NR	NR	NR
Number of patients gaining ≥ 15 letters, OR (95% CI)	4.22* (2.38, 7.47) [42]	CRUISE, 4.48* (2.52, 7.94) [42]	1.46 (0.76, 2.79) [18]	1.54 (0.79, 2.98) [18]	NR	NR	NR
Number of patients gaining ≥ 10 letters, OR (95% CI)	4.82* (2.84, 8.18) [42]	CRUISE, 7.12* (4.12, 12.29) [42]	NR	1.15 (0.67, 1.97) [18]	CVOS (12 months), 1.90 (0.65, 5.54) [10] Laatikainen et al (12 months), 1.00 (0.13, 7.75) [46] May et al (24 months), 0.70 (0.14, 3.56) [47]	NR	NR
*BRVO or CRVO*							
Number of patients gaining ≥ 10 letters, OR (95% CI)	NR	NR	NR	3 months, 9.82* (2.84, 33.99) [20]	NR	NR	NR

^a^Measured by Snellen chart score.

^b^Measured in LogMAR, ^c^The difference was reported as being not statistically significant (pooled data for BRVO and CRVO show significant improvement for mean difference in BCVA: OR 2.5 [95% CI 0.7, 4.3]).

*Statistically significant difference between groups.

*BCVA*, Best-corrected visual acuity; *BRVO*, Branch retinal vein occlusion; *BVOS*, Branch retinal Vein Occlusion Study; *CI*, Confidence interval; *CRUISE*, Ranibizumab for the Treatment of Macular Edema after Central Retinal Vein OcclUsIon Study: Evaluation of Efficacy and Safety; *CRVO,* Central retinal vein occlusion; *CVOS*, Central retinal Vein Occlusion Study; *Dex IVT*, Dexamethasone intravitreal; *ETDRS*, Early Treatment Diabetic Retinopathy Study; *IVB*, intravitreal bevacizumab; *logMAR*, Logarithm of minimum angle of resolution; *OR*, Odds ratio; *RBZ*, Ranibizumab; *ROCC*, Study Comparing Ranibizumab to Sham in Patients with Macular Edema Secondary to Central Retinal vein OCClusion; *NR*, Not reported; *NS*, Not significant.

### Efficacy

#### Ranibizumab

##### BRVO

The efficacy of ranibizumab 0.5 mg and 0.3 mg for treatment of BRVO has been investigated in a high-quality, double-blind, sham-controlled RCT, the BRAnch retinal Vein Occlusion: Evaluation of Efficacy and Safety (BRAVO) study, involving 397 patients [43]. The primary endpoint for the study was the mean change in BCVA at 6 months. Ranibizumab 0.5 mg produced greater improvements in BCVA at 6 months than sham and the difference was statistically and clinically significant (mean difference, 11.0 letters, 95% CI 7.83, 14.17; Table 4). The proportion of patients gaining at least 10 letters at 6 months was also significantly greater for ranibizumab 0.5 mg than for sham (OR 5.48, 95% CI 3.18, 9.44). Improvements in BCVA over sham and increases in the proportion of patients gaining at least 10 letters were both greater for ranibizumab 0.5 mg than for the 0.3 mg dose (Table 4).

In this study [43], patients not achieving sufficient improvement in BCVA could receive a single treatment with laser therapy after month 3 of the treatment period, as per standard of care. The proportion of patients who received laser therapy during the first 6 months was greater for the sham group than for the ranibizumab 0.5 mg group (55% vs. 20%).

##### CRVO

Two studies have investigated the efficacy of ranibizumab compared with sham injections in patients with CRVO [42,44]. A large, high-quality, double-blind, sham-controlled RCT, the Ranibizumab for the Treatment of Macular Edema after Central Retinal Vein OcclUsIon Study: Evaluation of Efficacy and Safety (CRUISE), assessed ranibizumab 0.5 mg and 0.3 mg in 392 patients [42]. A second smaller, double-blind, sham-controlled RCT, the randomized Study Comparing Ranibizumab to Sham in Patients with Macular Edema Secondary to Central Retinal vein OCClusion (ROCC), assessed ranibizumab 0.5 mg versus sham injection in 29 patients [44].

The primary endpoint for CRUISE was the mean change from baseline in BCVA at 6 months and the primary endpoints in ROCC were mean change from baseline in BCVA and central macular thickness at 6 months. In both studies, ranibizumab 0.5 mg produced greater improvements in BCVA than did sham at 6 months; the difference between ranibizumab and sham was clinically and statistically significant for CRUISE (mean difference 14.1, 95% CI 10.51, 17.69) but not for ROCC (mean difference 11.0, 95% CI –2.48, 24.48; Table 4). Analysis of pooled data from CRUISE and ROCC yielded a significant gain in mean BCVA for ranibizumab 0.5 mg over sham at 6 months (mean difference 13.89, 95% CI 10.42, 17.37) [42,44].

The proportion of patients gaining at least 10 letters at 6 months was also significantly greater for ranibizumab 0.5 mg than for sham in CRUISE (OR 7.12, 95% CI 4.12, 12.29; Table 4). Improvements in BCVA over sham and increases in the proportion of patients gaining at least 10 letters were both greater for ranibizumab 0.5 mg than for the 0.3 mg dose (Table 4).

#### Dexamethasone IVT

The efficacy of dexamethasone IVT in patients with BRVO or CRVO has been investigated in two large, high-quality, prospective, multicentre, masked, parallel-group RCTs in patients with BRVO or CRVO; the GENEVA studies [18,32]. These studies compared dexamethasone IVT 0.7 mg and 0.35 mg with sham injection. There was no rescue therapy for patients not responding to treatment. The trials collectively included 1267 patients and the prospectively defined primary endpoint for pooled data from the two studies was the time to reach a 15-letter improvement in BCVA from baseline. Data were reported as pooled data from the two studies.

##### BRVO

Significantly greater improvements in mean BCVA were achieved at 6 months with dexamethasone IVT 0.7 mg than with sham injections (mean difference 2.5 letters, 95% CI 0.6, 4.3, Table 4) [33]. The proportion of patients gaining at least 10 letters was also significantly greater for patients receiving dexamethasone IVT 0.7 mg than for sham at 6 months (OR 1.43, 95% CI 1.01, 2.01), although there was no significant difference in the proportion of patients gaining at least 15 letters at the same time point (OR 1.19, 95% CI 0.80, 1.78) [18]. Improvements in BCVA from baseline and in the proportion of patients gaining at least 10 and at least 15 letters with dexamethasone IVT 0.7 mg peaked at 2 months and declined thereafter.

##### CRVO

Significantly greater improvements in mean BCVA were achieved at 1, 2 and 3 months, but not at 6 months, with dexamethasone IVT 0.7 mg than with sham injections. The mean difference peaked at 2 months (9.3 letters, 95% CI 6.5, 12.1), decreased to 4.6 letters (95% CI 1.4, 7.8) at 3 months and was not statistically significant at 6 months [33]. Similarly, significant differences compared with sham were demonstrated for the proportion of patients gaining at least 15 letters at 1 and 2 months, and at least 10 letters at 1, 2 and 3 months, in patients receiving dexamethasone IVT 0.7 mg, but differences were not significant at 6 months for either endpoint (Table 4).

##### BRVO and CRVO

One further study compared dexamethasone IVT 0.35 mg and 0.7 mg with observation in patients with either BRVO or CRVO [20]. The study was a prospective, multicentre RCT that recruited 315 patients with macular oedema due to a variety of causes. Of these, 69 had RVO (not specified whether BRVO or CRVO) and were treated with dexamethasone IVT 0.7 mg; the percentage of patients gaining at least 10 letters at 3 months was significantly higher in patients receiving dexamethasone IVT (N = 35) than in those receiving sham (OR 9.82, 95% CI 2.84, 33.99).

#### Laser photocoagulation

##### BRVO

The efficacy of laser photocoagulation compared with no treatment/observation was assessed in patients with BRVO in two studies; one performed by the Branch Retinal Vein Occlusion Study (BVOS) group [6] and one by Battaglia Parodi *et al.*[45]. Both studies were intended to be masked, although 22% of the examinations at the 36-month time point in the BVOS had inadvertently become unmasked.

The BVOS, performed between 1977 and 1984, was a multicentre RCT involving 139 patients who received either laser therapy or no treatment [6]; the study had an average follow-up duration of 3.1 years. Data reported at 36 months showed that the proportion of patients gaining at least 10 letters was significantly greater in patients receiving laser therapy than in those receiving no treatment (OR 3.16, 95% CI 1.25, 8.00). The cumulative proportion of eyes that gained at least 10 letters since the initial visit for two or more consecutive visits increased throughout the study in both groups.

The second, more recent RCT for laser treatment involved 77 patients [45]. Patients received a single treatment of grid laser therapy at 3 months; at 9 months post-treatment, there was no significant difference between groups in mean change in BCVA from baseline (mean difference –0.01, 95% CI –0.08, 0.06).

##### CRVO

The Central Retinal Vein Occlusion Study (CVOS) group performed an RCT in 1995 involving 181 eyes [10]. Patients received either grid laser treatment or no treatment at baseline; the primary endpoint of the study was mean change in BCVA from baseline. The designs of the other two smaller studies were similar [46,47]. Patients were followed up every 3 months for assessment of BCVA and data are presented for 36 months for CVOS, 24 months for May *et al.* (1979) and 12 months for Laatikainen *et al.* (1977).

All three studies reported the percentage of patients gaining at least 10 letters; no statistically significant differences between treatment groups were observed in any of the studies at any time point assessed. In CVOS, there were also no significant differences between treatment groups in mean VA or the percentage of patients losing two lines by 36 months, and VA changed little in either group during the 36 months of follow-up.

#### Bevacizumab

##### BRVO

Two studies assessed the effects of bevacizumab in patients with BRVO; Moradian *et al.* compared bevacizumab with sham injections [49] and Russo *et al.* compared bevacizumab with laser therapy [50]; the dose of bevacizumab in both studies was 1.25 mg.

Moradian *et al.* performed a prospective RCT comparing bevacizumab and sham injections in 81 eyes with BRVO [49]; patients received two injections, one at baseline and one at 6 weeks. BCVA was measured using the Snellen chart and converted into logarithm of minimum angle of resolution (logMAR; lower score indicates better BCVA). At 6 weeks, patients treated with bevacizumab had a significantly greater improvement in BCVA from baseline than the sham group (mean difference [logMAR] –0.26, 95% CI –0.44, –0.08), although the difference was not significant at 12 weeks.

Russo *et al.* conducted an unmasked RCT in 30 eyes comparing bevacizumab versus laser therapy [50]. Patients received one treatment with laser therapy at baseline and another at 3 months if no improvement was observed. Patients treated with bevacizumab received one injection at baseline, then injections every 3 months until macular oedema resolved, as judged by optical coherence tomography. Mean BCVA was measured using logMAR. Improvements in BCVA at 6 months were statistically, although not clinically, significant for bevacizumab compared with laser therapy (mean difference [logMAR] –0.11, 95% CI –0.01, –0.21). The difference between groups in percentage of patients gaining at least 15 letters at the same time point was not statistically significant.

##### CRVO

Faghihi *et al.* (2008) performed a double-masked, multicentre RCT in 101 patients with CRVO receiving bevacizumab alone, bevacizumab plus IVTA or sham treatment [48]. At 18 weeks, BCVA (measured using logMAR) had improved in the bevacizumab-only group (by 0.47) and had worsened in the sham group (by 0.009); the difference between groups was statistically significant (P < 0.001). Error values were not reported for this study; hence, the mean difference and 95% CIs could not be calculated.

### Safety

Only three of the papers identified in this review reported detailed safety data; these provided results for ranibizumab 0.5 mg and 0.3 mg compared with sham injections (from BRAVO and CRUISE) [42,43] and dexamethasone IVT 0.7 mg and 0.35 mg compared with sham injections (the GENEVA studies) at 6 months (Table 5) [18,32]. No detailed safety data were reported in the laser therapy or bevacizumab studies.

**Table 5:** **Incidence of ocular AEs following treatment with ranibizumab [**[42][43]**] and dex IVT [**[18][32]**] at 6 months**

	**BRAVO [**[43]**]**^**a**^		**CRUISE [**[42]**]**^**a**^		**GENEVA**^**b **^**(BRVO and CRVO combined) [**[18]**,**[32]**]**	
**Adverse event**	**0.5 mg**	**Sham**	**0.5 mg**	**Sham**	**0.7 mg**	**Sham**
	**N = 130**	**N = 131**	**N = 129**	**N = 129**	**N = 421**	**N = 423**
Increased intraocular pressure^c^, N (%)	7 (5.4)	2 (1.5)	11 (8.5)	4 (3.1)	106 (25.2)^d^ OR 28.54, 95% CI 11.48, 70.95	5 (1.2)^d^
Ocular hypertension, N (%)	NR	NR	NR	NR	17 (4.0) P = 0.002	3 (0.7)
Eye pain, N (%)	21 (16.2)	19 (14.5)	24 (18.6)	13 (10.1)	31 (7.4)	16 (3.8)
Cataract, N (%)	4 (3.1)	4 (3.1)	2 (1.6)	0	31 (7.3)	19 (4.5)
Endophthalmitis, N (%)	1 (0.8)	0	0	0	NR	NR
Retinal detachment, N (%)	0	0	0	0	1	1
Iris neovascularization, N (%)	0	3 (2.3)	1 (0.8)	9 (7.0)	0	6 (1.4)
Retinal neovascularization, N (%)	0	5 (3.8)	2 (1.6)	3 (2.3)	3 (0.7) P = 0.032	11 (2.6)
Neovascular glaucoma, N (%)	0	0	0	2 (1.6)	NR	NR
Retinal tear, N (%)	0	0	0	0	NR	NR
Vitreous haemorrhage, N (%)	2 (1.5)	6 (4.6)	7 (5.4)	9 (7.0)	10 (2.4)	12 (2.8)
Retinal haemorrhage, N (%)	19 (14.6)	16 (12.2)	10 (7.8)	13 (10.0)	12 (2.9)	10 (2.4)

^a^Key study eye AEs through month 6.

^b^Ocular AEs in the study eye reported by > 2% of patients in any treatment group.

^c^Defined as ≥ 30 mmHg in BRAVO and CRUISE, and ≥ 25 mmHg in GENEVA.

^d^Data from the manufacturer’s submission to the National Institute for Health and Clinical Excellence (NICE).

*AE,* adverse event; *BRAVO*, Ranibizumab for the Treatment of Macular Edema after BRAnch retinal Vein Occlusion: Evaluation of Efficacy and Safety; *BRVO*, Branch retinal vein occlusion; *CRUISE*, Ranibizumab for the Treatment of Macular Edema after Central Retinal Vein OcclUsIon Study: Evaluation of Efficacy and Safety; *CRVO*, Central retinal vein occlusion; *dex IVT*, Dexamethasone intravitreal; *GENEVA*, Global EvaluatioN of implantable dExamethasone in retinal Vein occlusion with macular edemA; *NR*, Not reported.

The most notable difference between treatments was the incidence of increased intraocular pressure (IOP), which occurred in 25% of patients receiving dexamethasone IVT 0.7 mg. In addition, the proportion of patients requiring IOP-lowering therapy increased from 6% at baseline to 24% at 6 months in patients receiving dexamethasone IVT [18,32]. In contrast, the incidence of increased IOP was 5.4% and 8.5% in patients receiving ranibizumab 0.5 mg in BRAVO and CRUISE, respectively [42,43]. The incidence of cataract was low following treatment with ranibizumab 0.5 mg in both studies (< 4%) and was slightly elevated in patients receiving dexamethasone IVT 0.7 mg compared with sham (7.3% vs. 4.5%), although the difference was not significant. No cases of endophthalmitis were observed with dexamethasone IVT 0.7 mg; one case was observed in BRAVO in a patient receiving ranibizumab 0.5 mg. Retinal detachment was reported in one patient receiving sham and one receiving dexamethasone IVT in the GENEVA studies; there were no cases in BRAVO or CRUISE.

### Feasibility of indirect comparisons

The main purpose of this review was to compare ranibizumab with other available treatments for RVO in the UK. The feasibility of performing indirect comparisons between ranibizumab and the other treatments was therefore assessed by considering the available studies and their homogeneity according to detailed guidance from the Australian Pharmaceutical Benefits Advisory Committee [41] and additional guidance from NICE [51].

**Figure 2:**
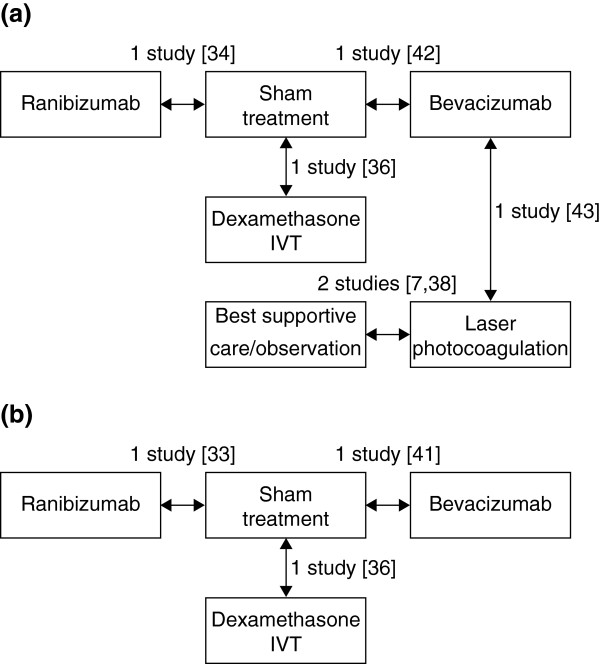
**Potential comparisons between ranibizumab and other treatments for (a) BRVO and (b) CRVO.** BRVO, branch retinal vein occlusion; CRVO, central retinal vein occlusion; IVT, intravitreal triamcinolone.

Figure 2a shows the potential indirect comparisons that could be made for ranibizumab with the other treatments for BRVO based on the identified literature. Comparisons need to be made via sham as this was the comparator in the only study of ranibizumab in BRVO (i.e. BRAVO) [43]. However, in this study, patients not achieving sufficient improvement in BCVA could receive laser therapy as in current clinical practice, which potentially underestimates the benefit of ranibizumab in relation to other treatments (Table 6). Other aspects of study design that add bias to indirect comparisons include: 1) the Moradian and Battaglia Parodi studies involved fewer than 100 patients compared with 397 patients in BRAVO, which would affect the precision of the effect estimate that would be captured in an indirect comparison; 2) the duration of follow-up was shorter in the Moradian study than in BRAVO (3 months vs. the 6-month primary endpoint in BRAVO) and was much longer in one of the laser studies (36 months), and 3) the two laser studies were not blinded. Furthermore, the duration of macular oedema at baseline was shorter in BRAVO than GENEVA (3.3–3.7 months vs. 5.1–5.3 months across treatment groups, respectively), which potentially underestimates the benefits of dexamethasone IVT compared with ranibizumab. It should be noted that duration of RVO in the GENEVA studies was calculated at the baseline visit. Conversely, in BRAVO and CRUISE, the duration of macular oedema was calculated at screening, up to 28 days before the baseline visit, which reduces the potential underestimation of treatment benefits of dexamethasone IVT over ranibizumab. In assessing feasibility of indirect comparisons, the duration of macular oedema before treatment was still considered important enough to hinder a robust analysis. Overall, the magnitude and direction of these sources of bias are difficult to determine. We therefore conclude that robust indirect comparisons of active treatments for BRVO are not feasible from the available published literature.

**Table 6:** Summary of similarities and differences between studies

**Studies**	**Similarities**	**Differences**
*BRVO*		
Ranibizumab vs. dex IVT: BRAVO [43] vs. GENEVA [18]	Design: double-blind	Patients failing to achieve sufficient improvement in BCVA could receive laser in BRAVO
	Date of study	Duration of macular oedema before study commencement: 3.3–3.7 months vs. 5.1–5.3 months across treatment groups for BRAVO and GENEVA, respectively
	Duration of follow-up:	
	6 months	
	Size	
	Patient demographics	
Ranibizumab vs. bevacizumab: BRAVO [43] vs. Moradian [49]	Design: double-blind	Patients failing to achieve sufficient improvement in BCVA could receive laser therapy in BRAVO
	Date of study	Size: 81 patients (Moradian) vs. 397 patients (BRAVO)
		Patient age: 58 years (Moradian) vs. 66 years (BRAVO)
		Duration of macular oedema at baseline: 6 weeks (Moradian) vs. 3.5 months (BRAVO)
		Duration of follow-up: 3 months (Moradian) vs. 6 months (BRAVO)
		BCVA endpoint: change in logMAR reported for Moradian
Ranibizumab vs. laser: BRAVO [43] vs. BVOS [6]	Patient demographics	Patients failing to achieve sufficient improvement in BCVA could receive laser therapy in BRAVO
	Size	Design: double-blinded for BRAVO but single-blinded for BVOS (patients were aware of their treatment)
		Duration of follow-up: 6 months vs. 36 months
Ranibizumab vs. laser: BRAVO [43] vs. Battaglia Parodi [45]	Patient demographics	Patients failing to achieve sufficient improvement in BCVA could receive laser in BRAVO
		Size: 77 patients (Battaglia Parodi) vs. 397 patients (BRAVO)
		Design: blinded for BRAVO but not reported for Battaglia Parodi
		Duration of follow-up: 24 months (Battaglia Parodi) vs. 6 months (BRAVO)
		Duration of BRVO: < 15 days for Battaglia Parodi vs. < 12 months for BRAVO (inclusion criterion)
*CRVO*		
Ranibizumab vs. dex IVT: CRUISE [42] vs. GENEVA [18]	Design: double-blind	Duration of macular oedema before study commencement: 2.9–3.6 months vs. 5.1–5.3 months across treatment groups for CRUISE and GENEVA, respectively
	Date of study	
	Duration of follow-up: 6 months	Baseline BCVA: 47.4–49.2 letters vs. 53.9–54.8 letters across treatment groups for CRUISE and GENEVA, respectively
	Size	
	Patient demographics	
Ranibizumab vs. laser: CRUISE [42] vs. CVOS [10]	Size	Design: blinded for CRUISE but single-blinded for CVOS (patients were aware of their treatment)
	Patient demographics	Duration of macular oedema at baseline: > 3 months for CVOS, < 12 months for CRUISE (inclusion criteria)
		Duration of follow-up: 36 months (CVOS) vs. 6 months (CRUISE)
Ranibizumab vs. laser: CRUISE [42] vs. Laatikainen [46]	Patient demographics	Size: 48 patients (Laatikainen) vs. 392 patients (CRUISE)
		Design: blinded for CRUISE but not Laatikainen
		Duration of follow-up: 12 months (Laatikainen) vs. 6 months (CRUISE)
		Duration of macular oedema at baseline: < 3 months for Laatikainen vs. < 12 months for CRUISE (inclusion criterion)
Ranibizumab vs. laser: CRUISE [42] vs. May [47]	Patient demographics	Size: 34 patients (May) vs. 392 patients (CRUISE)
		Design: blinded for CRUISE but not May
		Duration of follow-up: 24 months (May) vs. 6 months (CRUISE)
		CRVO duration at baseline: not specified for May vs. < 12 months for CRUISE (inclusion criterion)

*BCVA*, Best corrected visual acuity; *BRAVO*, Ranibizumab for the Treatment of Macular Edema after BRAnch retinal Vein Occlusion: Evaluation of Efficacy and Safety; *BRVO*, Branch retinal vein occlusion; *BVOS*, Branch retinal Vein Occlusion Study; *CRUISE*, Ranibizumab for the Treatment of Macular Edema after Central Retinal Vein OcclUsIon Study: Evaluation of Efficacy and Safety; *CRVO*, Central retinal vein occlusion; *CVOS*, Central retinal Vein Occlusion Study; *Dex IVT*, Dexamethasone intravitreal; *GENEVA*, Global EvaluatioN of implantable dExamethasone in retinal Vein occlusion with macular edemA; *logMAR*, Logarithm of minimum angle of resolution; *NR*, Not reported.

Figure 2b shows the potential indirect comparisons that could be made for ranibizumab with the other treatments for CRVO. All comparisons needed to be made via sham, the comparator in CRUISE. Although there is a second sham-controlled study of ranibizumab, ROCC, this smaller study did not report the proportion of patients gaining at least 15 letters, the outcome measure common to all the other studies, and hence cannot be considered for this analysis. Comparison via sham was considered for ranibizumab versus dexamethasone IVT based on data from the GENEVA study. As observed for the comparison for BRVO, the difference in duration of macular oedema before study commencement between CRUISE and GENEVA (2.9–3.6 months vs. 5.1–5.3 months across treatment groups, respectively) may underestimate the benefits of dexamethasone IVT compared with ranibizumab, although the impact of this is reduced when the additional screening period in CRUISE is taken into account (Table 6). Furthermore, baseline BCVA was lower in CRUISE than GENEVA (47.4–49.2 letters vs. 53.9–54.8 letters across treatment groups, respectively), adding bias towards ranibizumab. Owing to uncertainties in the overall magnitude of bias, we deemed indirect comparison between ranibizumab and dexamethasone for the treatment of CRVO unfeasible. One study that compared bevacizumab with sham was identified [48]. However, standard errors were not reported for this study, thus preventing performance of an indirect comparison between ranibizumab and bevacizumab in patients with CRVO.

## Discussion

For several decades, laser therapy was the standard of care for patients with BRVO in the UK [4]. This is largely based on the results of the BVOS, conducted over 30 years ago, which reported that significantly more patients receiving laser therapy had gained at least 10 letters at 36 months than patients receiving no treatment [6]. However, differences in the improvement in VA between the two treatment groups became apparent only after 12 months, and 12% of patients treated with laser therapy experienced decreases in VA during the study, as judged by losing at least two letters at two consecutive visits during the study. More recent data from the Standard Care vs. Corticosteroid for Retinal Vein Occlusion (SCORE) study also show limited benefit for laser therapy at 12 months (median gain of six letters at 12 months) [23]. This is further supported by the fact that a more recent but smaller study reported no benefit of laser therapy compared with no treatment at 9 months [45]. In contrast, three studies showed no benefit of laser therapy for the treatment of CRVO, and laser therapy is not recommended for this indication [10,46,47]. Ranibizumab and dexamethasone IVT implant have recently been approved for treatment of visual impairment due to macular oedema secondary to both BRVO and CRVO. It is therefore relevant to assess their relative efficacy and safety, and to compare them with other therapies.

Results from two large, high-quality, double-blind, sham-controlled RCTs (BRAVO and CRUISE) reported ranibizumab to be an effective treatment for both BRVO and CRVO [42,43]. In both indications, ranibizumab 0.5 mg induced statistically and clinically significant improvements in BCVA and in the proportion of patients gaining at least 15 letters at 6 months. Furthermore, statistically significant differences in the change in BCVA from baseline between ranibizumab and sham were evident from day 7 onwards in both studies. Supporting evidence for the effectiveness of ranibizumab comes from a smaller, double-blind, sham-controlled study in patients with CRVO (ROCC) [44], which also reported statistically and clinically significant differences between ranibizumab 0.5 mg and sham for improvement in BCVA at 6 months. It should be noted that patients not achieving sufficient improvements at month 3 in BRAVO in either group could receive laser therapy, thus possibly confounding the results from month 3 onwards. However, the BVOS and SCORE studies showed that the benefits of laser therapy are minimal within the first year of treatment [6]; hence, the benefit of laser therapy in either treatment group may not be evident at the 6-month time point.

The GENEVA studies reported that dexamethasone IVT 0.7 mg induced significant improvements in mean BCVA at 6 months in patients with BRVO, although the difference was not significant in patients with CRVO [18]. In both indications, there was no significant difference between groups for the proportion of patients gaining at least 15 letters at 6 months. However, significant differences for this endpoint were demonstrated at months 1, 2 and 3 in patients with BRVO, and at months 1 and 2 for patients with CRVO. In both indications, improvements in the proportion of patients gaining at least 15 letters peaked at 2 months and declined thereafter, suggesting that the duration of benefit is not sustained beyond 3 months following administration of a single implant of dexamethasone IVT 0.7 mg.

Results from open-label extension studies to BRAVO, CRUISE and GENEVA (not included in this review as they were not RCTs) have been reported and provide further data on the efficacy and safety of ranibizumab up to 24 months and dexamethasone IVT up to 12 months [52-55]; in the extensions to BRAVO and CRUISE, all patients received ranibizumab 0.5 mg on an as-needed basis. These studies reported that improvements in BCVA following 6 months of treatment with ranibizumab were maintained up to 24 months in patients with BRVO [52,55] and up to 12 months in patients with CRVO, followed by a slight loss in BCVA between months 12 and 24 [53,55]. In the extension to the GENEVA studies, patients received an implant of dexamethasone IVT 0.7 mg at day 180 (i.e. a second implant for those who received dexamethasone IVT at the start of GENEVA and a first implant for those treated with sham in GENEVA) [54]. The response to the second treatment was similar to that observed with the first treatment and the improvement in BCVA from baseline peaked at 2 months after administration of the implant and declined thereafter.

In contrast to ranibizumab and dexamethasone IVT, evidence for the efficacy of bevacizumab in BRVO and CRVO is limited. This systematic review identified only two small RCTs in BRVO [49,50] and a single RCT in CRVO, each involving fewer than 100 patients. Statistically significant differences in improvement in BCVA at 1, 3, 6 and 12 months compared with laser therapy were reported in one study in BRVO [50] and the second study reported significantly greater improvements in BCVA at 6 weeks for bevacizumab than for no treatment, although the difference was not significant at 12 weeks [49]. A third study in CRVO reported an improvement in BCVA for bevacizumab and bevacizumab plus IVTA at 18 weeks compared with a decrease in BCVA for sham. Longer follow-up of larger numbers of patients is required to assess meaningfully the potential benefit of bevacizumab in this indication.

Results of the BRAVO, CRUISE and GENEVA studies provide an assessment of the safety profile of ranibizumab and dexamethasone IVT in patients with RVO. Ranibizumab was generally well tolerated in BRAVO and CRUISE [42,43], and the follow-up studies show that this favourable safety profile was maintained in the open-label extensions [52,53,55]. These findings are consistent with the safety profile observed for ranibizumab in other ocular conditions, including a low incidence of endophthalmitis, few ocular SAEs, and low rates of adverse systemic cardiovascular and cerebrovascular effects (for which there is an increased theoretical risk when using anti-VEGF agents) [11,56-58]. Results from the GENEVA study indicate that dexamethasone IVT 0.7 mg is associated with an increased risk of developing elevated IOP at 6 months (occurred in 25.2% of eyes) [18], which increased to 32.8% at 12 months in patients who received two dexamethasone IVT 0.7 mg implants [54]. The rate of cataract was increased compared with controls at 6 months (7.3%), although the difference was not significant; at 12 months, the rate of cataract in patients receiving two implants had increased to 29.8%. This is in agreement with the known safety profile of corticosteroids as evident in the SCORE study, which reported a significantly higher incidence of elevated IOP and cataract for IVTA than for laser therapy (in BRVO) or observation (in CRVO) [23]. Given that retreatment with dexamethasone IVT may well occur after 4 months in clinical practice, rather than 6 months as in the GENEVA studies [40], the incidence of elevated IOP and cataract with dexamethasone IVT may well be higher in routine practice and is likely to require additional monitoring [56-58]. In contrast to ranibizumab and dexamethasone IVT, the safety profile of bevacizumab in RVO has not been rigorously assessed. The incidence of AEs was not reported for two of the bevacizumab studies [48,49], and the third study [50] reported minor local adverse reactions related to treatment with bevacizumab in 9 out of 15 patients during the first week following the first injection. Bevacizumab is unlicensed for ocular use and further detailed safety data and pharmacovigilance in RVO are essential. Safety data were not rigorously assessed in any of the studies of laser therapy. However, laser therapy is widely used in various ocular indications and is known to be associated with various complications, including foveal burns, central visual field defects, exacerbation of macular oedema and acute glaucoma [59-61].

This review included published data for treatments that were widely used and available at the time of the study (November 2010). Since this systematic review was undertaken, results from two large double-blind, sham controlled RCTs (COPERNICUS and GALILEO) have been reported and provide data on the efficacy and safety of another anti-VEGF, aflibercept, in CRVO [62-65]. These studies suggest that monthly injections of aflibercept result in significant improvements in visual acuity at month 6 compared with sham injections, and that improvements were sustained for up to 12 months with further injections administered *pro re nata*. Aflibercept was generally well tolerated, with a low incidence of ocular SAEs [62-65]. Data on the efficacy and safety of aflibercept would warrant inclusion in a future systematic review.

## Conclusions

In conclusion, data from high-quality RCTs for ranibizumab and dexamethasone IVT have demonstrated that both new agents have promising outcomes for the treatment of BRVO and CRVO, and constitute significant improvements over the previously widely accepted standards of care (laser therapy for BRVO and no treatment for CRVO). Significant differences in study design and patient baseline characteristics prevent indirect comparisons being made between these treatments. Ranibizumab and dexamethasone IVT both produce rapid improvements in BCVA. These improvements appear to be maintained with initial monthly therapy followed by treatment as needed for ranibizumab, but decline 3 months after administration of the dexamethasone IVT implant. Dexamethasone IVT is associated with a significantly greater risk of increased IOP (which increased further with repeated treatment) and possibly an increased risk of cataract than is sham.

Head-to-head comparative studies are urgently needed to assess the relative efficacies of available licensed therapies for RVO. This is currently being assessed in three ongoing RCTs comparing ranibizumab with dexamethasone IVT in patients with RVO; the COMO (NCT01427751) and COMRADE B (NCT01396057) studies are being conducted in patients with BRVO and the COMRADE C study (NCT01396083) is being conducted in patients with CRVO. Direct comparison of the licensed therapies with laser monotherapy and the role of adjunctive laser therapy are currently lacking. This issue is being addressed by the RABAMES study (NCT00562406) comparing ranibizumab, laser monotherapy and ranibizumab plus adjunctive laser therapy in patients with BRVO; this study has been completed and data are expected shortly. A further study comparing similar treatment arms, the BRIGHTER study (NCT01599650, EUDRACT 2011-002859-34), has begun recruiting in Europe. Results from these studies should help to clarify the roles of the licensed therapies in the management of patients with RVO.

## Abbreviations

AE: Adverse event; BCVA: Best corrected visual acuity; BRVO: Branch retinal vein occlusion; CI: Confidence interval; CRVO: Central retinal vein occlusion; IOP: Intraocular pressure; IVT: Intravitreal; IVTA: Intravitreal triamcinolone; logMAR: Logarithm of minimum angle of resolution; OR: Odds ratio; RCT: Randomized controlled trial; RVO: Retinal vein occlusion; SAE: Serious adverse event; VA: Visual acuity; VEGF: Vascular endothelial growth factor.

## Competing interests

Alberto Ferreira and Kerry Gairy are both employees and stakeholders of the Novartis Group. Julie Glanville, Jacoby Patterson and Rachael McCool received funding from Novartis to carry out reviews and generate economic models. Ian Pearce acts as a consultant and lecturer for Novartis.

## Authors’ contributions

Study protocol was developed by JG, AF and KG. All authors participated in the development and writing of the manuscript, and approved the final article for publication. JG, JP and RM also performed screening, data extraction and synthesis, and assessment of feasibility of indirect comparisons.

## Pre-publication history

The pre-publication history for this paper can be accessed here:

http://www.biomedcentral.com/1471-2415/14/7/prepub

## Supplementary Material

Additional file 1 Database search strategy for MEDLINE and MEDLINE In-Process. Click here for file

Additional file 2 Assessment of study quality and risk of bias. Click here for file

## References

[B1] Parodi MB Bandello F Branch retinal vein occlusion: classification and treatment Ophthalmologica 2009 223 5 298 305 10.1159/000213640 19372724

[B2] Hayreh SS Zimmerman MB Podhajsky P Incidence of various types of retinal vein occlusion and their recurrence and demographic characteristics Am J Ophthalmol 1994 117 4 429 441 8154523 10.1016/s0002-9394(14)70001-7

[B3] Wong TY Scott IU Clinical practice. Retinal-vein occlusion N Engl J Med 2010 363 22 2135 2144 10.1056/NEJMcp1003934 21105795

[B4] Royal College of Ophthalmologists Retinal vein occlusion (RVO): interim guidelines for management of retinal vein occlusion (RVO) 2010 London: RCOpth Available from: http://www.rcophth.ac.uk/core/core_picker/download.asp?id=337

[B5] Yau JW Lee P Wong TY Best J Jenkins A Retinal vein occlusion: an approach to diagnosis, systemic risk factors and management Intern Med J 2008 38 12 904 910 10.1111/j.1445-5994.2008.01720.x 19120547

[B6] BVOS Argon laser photocoagulation for macular edema in branch vein occlusion. The branch vein occlusion study group Am J Ophthalmol 1984 98 3 271 282 6383055 10.1016/0002-9394(84)90316-7

[B7] McIntosh RL Rogers SL Lim L Cheung N Wang JJ Mitchell P Kowalski JW Nguyen HP Wong TY Natural history of central retinal vein occlusion: an evidence-based systematic review Ophthalmology 2010 117 6 1113 1123 e1115 10.1016/j.ophtha.2010.01.060 20430446

[B8] Awdeh RM Elsing SH Deramo VA Stinnett S Lee PP Fekrat S Vision-related quality of life in persons with unilateral branch retinal vein occlusion using the 25-item national eye institute visual function questionnaire Br J Ophthalmol 2010 94 3 319 323 10.1136/bjo.2007.135913 19737736

[B9] Deramo VA Cox TA Syed AB Lee PP Fekrat S Vision-related quality of life in people with central retinal vein occlusion using the 25-item national eye institute visual function questionnaire Arch Ophthalmol 2003 121 9 1297 1302 10.1001/archopht.121.9.1297 12963613

[B10] CVOS Evaluation of grid pattern photocoagulation for macular edema in central vein occlusion. The central vein occlusion study group Ophthalmology 1995 102 10 1425 1433 9097788 10.1016/s0161-6420(95)30849-4

[B11] Varma R Wu J Chong K Azen SP Hays RD Impact of severity and bilaterality of visual impairment on health-related quality of life Ophthalmology 2006 113 10 1846 1853 10.1016/j.ophtha.2006.04.028 16889831

[B12] Ozurdex summary of product characteristics last updated December 2011 Available at: http://www.medicines.org.uk/emc/medicine/23422/SPC/ozurdex/. Accessed July 2012

[B13] Lucentis prescribing information (US) last updated June 2010 Available at: http://www.gene.com/gene/products/information/pdf/lucentis-prescribing.pdf. Accessed July 2012

[B14] Ozurdex prescribing information (US) last updated February 2012 Available at: http://www.allergan.com/assets/pdf/ozurdex_pi.pdf. Accessed July 2012

[B15] Pieramici DJ Rabena M Castellarin AA Nasir M See R Norton T Sanchez A Risard S Avery RL Ranibizumab for the treatment of macular edema associated with perfused central retinal vein occlusions Ophthalmology 2008 115 10 e47 54 10.1016/j.ophtha.2008.06.021 18708258

[B16] McAllister IL Vijayasekaran S Chen SD Yu DY Effect of triamcinolone acetonide on vascular endothelial growth factor and occludin levels in branch retinal vein occlusion Am J Ophthalmol 2009 147 5 838 846 e831-832 10.1016/j.ajo.2008.12.006 19211093

[B17] Leopold IH Sears ML, Tarkkanen A Nonsteroidal and steroidal anti-inflammatory agents Surgical Pharmacology of the Eye 1985 New York: Raven Press 83 133

[B18] Haller JA Bandello F Belfort R Jr Blumenkranz MS Gillies M Heier J Loewenstein A Yoon YH Jacques ML Jiao J Randomized, sham-controlled trial of dexamethasone intravitreal implant in patients with macular edema due to retinal vein occlusion Ophthalmology 2010 117 6 1134 1146 e1133 10.1016/j.ophtha.2010.03.032 20417567

[B19] Koss MJ Pfister M Rothweiler F Michaelis M Cinatl J Schubert R Koch FH Comparison of cytokine levels from undiluted vitreous of untreated patients with retinal vein occlusion Acta Ophthalmol 2012 90 2 e98 e103 10.1111/j.1755-3768.2011.02292.x 22066978

[B20] Kuppermann BD Blumenkranz MS Haller JA Williams GA Weinberg DV Chou C Whitcup SM Randomized controlled study of an intravitreous dexamethasone drug delivery system in patients with persistent macular edema Arch Ophthalmol 2007 125 3 309 317 10.1001/archopht.125.3.309 17353400

[B21] Pfister M Rothweiler F Michaelis M Cinatl J Jr Schubert R Koch FH Koss MJ Correlation of inflammatory and proangiogenic cytokines from undiluted vitreous samples with spectral domain OCT scans, in untreated branch retinal vein occlusion Clin Ophthalmol 2013 7 1061 1067 23766628 10.2147/OPTH.S42786 PMC3677846

[B22] Wang K Wang Y Gao L Li X Li M Guo J Dexamethasone inhibits leukocyte accumulation and vascular permeability in retina of streptozotocin-induced diabetic rats via reducing vascular endothelial growth factor and intercellular adhesion molecule-1 expression Biol Pharm Bull 2008 31 8 1541 1546 10.1248/bpb.31.1541 18670086

[B23] Scott IU Ip MS VanVeldhuisen PC Oden NL Blodi BA Fisher M Chan CK Gonzalez VH Singerman LJ Tolentino M A randomized trial comparing the efficacy and safety of intravitreal triamcinolone with standard care to treat vision loss associated with macular edema secondary to branch retinal vein occlusion: the Standard Care vs Corticosteroid for Retinal Vein Occlusion (SCORE) study report 6 Arch Ophthalmol 2009 127 9 1115 1128 19752420 10.1001/archophthalmol.2009.233 PMC2806600

[B24] Zhang X Bao S Lai D Rapkins RW Gillies MC Intravitreal triamcinolone acetonide inhibits breakdown of the blood-retinal barrier through differential regulation of VEGF-A and its receptors in early diabetic rat retinas Diabetes 2008 57 4 1026 1033 10.2337/db07-0982 18174522 PMC2836241

[B25] Eylea prescribing information (US) last updated September 2012 Available at: http://www.regeneron.com/Eylea/eylea-fpi.pdf. Accessed July 2012

[B26] Center for Reviews and Dissemination Systemtic Reviews: CRD’s guidance for undertaking reviews in health care 2009 York: CRD, University of York

[B27] Gray S Rubio R A phase III, multicenter, randomized, sham injection-controlled study of the efficacy and safety of ranibizumab injection compared with sham in subjects with macular edema secondary to branch retinal vein occlusion [Clinical Study Report] 2009 Genentech: South San Francisco, CA

[B28] Gray S Rubio R A phase III, multicenter, randomized, sham injection-controlled study of the efficacy and safety of ranibizumab injection compared with sham in subjects with macular edema secondary to branch retinal vein occlusion [Clinical Study Report Addendum] 2009 Genentech: South San Francisco, CA

[B29] Gray S Rubio R A phase III, multicenter, randomized, sham injection-controlled study of the efficacy and safety of ranibizumab injection compared with sham in subjects with macular edema secondary to central retinal vein occlusion [Clinical Study Report] 2010 Genentech: South San Francisco, CA

[B30] Gray S Rubio R A phase III, multicenter, randomized, sham injection-controlled study of the efficacy and safety of ranibizumab injection compared with sham in subjects with macular edema secondary to central retinal vein occlusion [Clinical Study Report Addendum] 2010 Genentech: South San Francisco, CA

[B31] Singer M Gray S Yee Murahashi W Saroj N Rundle A Rubio R Subgroup analyses of visual acuity outcomes in the bravo study of intravitreal ranibizumab in patients with macular edema following branch retinal vein occlusion ARVO Meeting Abstracts 2010 51 3561 Available from: http://abstracts.iovs.org//cgi/content/abstract/51/5/3561

[B32] Shyangdan D Cummins E Lois N Royle P Waugh N Dexamethasone implants (Ozurdex) for macular oedema after retinal vein occlusion. Single technology appraisal (STA) 2010 AbHTAG Available from: http://www.nice.org.uk/nicemedia/live/13037/52883/52883.pdf

[B33] Allergan Dexamethasone intravitreal implant (Ozurdex®) for the treatment of macular oedema caused by retinal vein occlusion. Single technology appraisal (STA) 2010 Allergan Available from: http://www.nice.org.uk/nicemedia/live/13037/52863/52863.pdf

[B34] Allergan A study of the safety and efficacy of a new treatment for macular edema resulting from retinal vein occlusion. NCT00168298 2009 Available from: http://ClinicalTrials.gov/show/NCT00168298

[B35] Allergan A Study of the Safety and Efficacy of a New Treatment for Macular Edema Resulting From Retinal Vein Occlusion. NCT00168324 2009 Available from: http://ClinicalTrials.gov/show/NCT00168324

[B36] Chang TS Bressler NM Fine JT Dolan CM Ward J Klesert TR Improved vision-related function after ranibizumab treatment of neovascular age-related macular degeneration: results of a randomized clinical trial Arch Ophthalmol 2007 125 11 1460 1469 10.1001/archopht.125.11.1460 17998507

[B37] Mangione CM Lee PP Gutierrez PR Spritzer K Berry S Hays RD Development of the 25-item national eye institute visual function questionnaire Arch Ophthalmol 2001 119 7 1050 1058 10.1001/archopht.119.7.1050 11448327

[B38] Margolis MK Coyne K Kennedy-Martin T Baker T Schein O Revicki DA Vision-specific instruments for the assessment of health-related quality of life and visual functioning: a literature review Pharmacoeconomics 2002 20 12 791 812 10.2165/00019053-200220120-00001 12236802

[B39] Csaky KG Richman EA Ferris FL 3rd Report from the NEI/FDA ophthalmic clinical trial design and endpoints symposium Invest Ophthalmol Vis Sci 2008 49 2 479 489 10.1167/iovs.07-1132 18234989

[B40] NICE Single technology appraisal (STA). Specification for manufacturer/sponsor submission of evidence 2009 London: NICE

[B41] PBAC Report of the Indirect Comparisons Working Group to the Pharmaceutical Benefits Advisory Committee: assessing indirect comparisons 2008 PBAC Available from: http://www.pbs.gov.au/industry/useful-resources/PBAC_feedback_files/ICWG%20Report%20FINAL2.pdf

[B42] Brown DM Campochiaro PA Singh RP Li Z Gray S Saroj N Rundle AC Rubio RG Murahashi WY Ranibizumab for macular edema following central retinal vein occlusion: six-month primary end point results of a phase III study Ophthalmology 2010 117 6 1124 1133 e1121 10.1016/j.ophtha.2010.02.022 20381871

[B43] Campochiaro PA Heier JS Feiner L Gray S Saroj N Rundle AC Murahashi WY Rubio RG Ranibizumab for macular edema following branch retinal vein occlusion: six-month primary end point results of a phase III study Ophthalmology 2010 117 6 1102 1112 e1101 10.1016/j.ophtha.2010.02.021 20398941

[B44] Kinge B Stordahl PB Forsaa V Fossen K Haugstad M Helgesen OH Seland J Stene-Johansen I Efficacy of ranibizumab in patients with macular edema secondary to central retinal vein occlusion: results from the sham-controlled ROCC study Am J Ophthalmol 2010 150 3 310 314 10.1016/j.ajo.2010.03.028 20591399

[B45] Battaglia Parodi M Saviano S Bergamini L Ravalico G Grid laser treatment of macular edema in macular branch retinal vein occlusion Doc Ophthalmol 1999 97 3–4 427 431 10896360 10.1023/a:1002452004743

[B46] Laatikainen L Kohner EM Khoury D Blach RK Panretinal photocoagulation in central retinal vein occlusion: a randomised controlled clinical study Br J Ophthalmol 1977 61 12 741 753 10.1136/bjo.61.12.741 341965 PMC1043112

[B47] May DR Klein ML Peyman GA Raichand M Xenon arc panretinal photocoagulation for central retinal vein occlusion: a randomised prospective study Br J Ophthalmol 1979 63 11 725 734 10.1136/bjo.63.11.725 508687 PMC1043608

[B48] Faghihi H Piri N Esfahani M Aalami Z Lashay A Piri N Faghihi S Intravitreal bevacizumab vs. combination of bevacizumab and triamcinolone vs. sham treatment in Central Retinal Vein Occlusion 2008 American Academy of Ophthalmology Meeting Archives 271 Available from: http://aao.scientificposters.com/epsView.cfm?8o86zyfE5SMw0%2FURIHEaEPee1tSQzRJzYw6PAGSTN9mNNuonO4x%2FYg%3D%3D

[B49] Moradian S Faghihi H Sadeghi B Piri N Ahmadieh H Soheilian M Dehghan MH Azarmina M Esfahani MR Intravitreal bevacizumab vs. sham treatment in acute branch retinal vein occlusion with macular edema: results at 3 months (Report 1) Graefes Arch Clin Exp Ophthalmol 2011 249 2 193 200 10.1007/s00417-010-1440-8 21337043

[B50] Russo V Barone A Conte E Prascina F Stella A Noci ND Bevacizumab compared with macular laser grid photocoagulation for cystoid macular edema in branch retinal vein occlusion Retina 2009 29 4 511 515 10.1097/IAE.0b013e318195ca65 19174717

[B51] The National Institute for Health and Clinical Excellence Guide to the methods of technology appraisal last updated June 2008 2012 Available at: http://www.nice.org.uk/media/B52/A7/TAMethodsGuideUpdatedJune2008.pdf 27905712

[B52] Brown DM Campochiaro PA Bhisitkul RB Ho AC Gray S Saroj N Adamis AP Rubio RG Murahashi WY Sustained benefits from ranibizumab for macular edema following branch retinal vein occlusion: 12-month outcomes of a phase III study Ophthalmology 2011 118 8 1594 1602 10.1016/j.ophtha.2011.02.022 21684606

[B53] Campochiaro PA Brown DM Awh CC Lee SY Gray S Saroj N Murahashi WY Rubio RG Sustained benefits from ranibizumab for macular edema following central retinal vein occlusion: twelve-month outcomes of a phase III study Ophthalmology 2011 118 10 2041 2049 10.1016/j.ophtha.2011.02.038 21715011

[B54] Haller JA Bandello F Belfort R Jr Blumenkranz MS Gillies M Heier J Loewenstein A Yoon YH Jiao J Li XY Dexamethasone intravitreal implant in patients with macular edema related to branch or central retinal vein occlusion twelve-month study results Ophthalmology 2011 118 12 2453 2460 10.1016/j.ophtha.2011.05.014 21764136

[B55] Heier JS Campochiaro PA Yau L Li Z Saroj N Rubio RG Lai P Ranibizumab for macular edema due to retinal vein occlusions long-term follow-up in the HORIZON trial Ophthalmology 2012 119 4 802 809 10.1016/j.ophtha.2011.12.005 22301066

[B56] Brown DM Kaiser PK Michels M Soubrane G Heier JS Kim RY Sy JP Schneider S Ranibizumab versus verteporfin for neovascular age-related macular degeneration N Engl J Med 2006 355 14 1432 1444 10.1056/NEJMoa062655 17021319

[B57] Elman MJ Aiello LP Beck RW Bressler NM Bressler SB Edwards AR Ferris FL III Friedman SM Glassman AR Miller KM Randomized trial evaluating ranibizumab plus prompt or deferred laser or triamcinolone plus prompt laser for diabetic macular edema Ophthalmology 2010 117 6 1064 1077 e1035 10.1016/j.ophtha.2010.02.031 20427088 PMC2937272

[B58] Regillo CD Brown DM Abraham P Yue H Ianchulev T Schneider S Shams N Randomized, double-masked, sham-controlled trial of ranibizumab for neovascular age-related macular degeneration: PIER Study year 1 Am J Ophthalmol 2008 145 2 239 248 10.1016/j.ajo.2007.10.004 18222192

[B59] Aiello LM Perspectives on diabetic retinopathy Am J Ophthalmol 2003 136 1 122 135 10.1016/S0002-9394(03)00219-8 12834680

[B60] Lovestam-Adrian M Agardh E Photocoagulation of diabetic macular oedema complications and visual outcome Acta Ophthalmol Scand 2000 78 6 667 671 10.1034/j.1600-0420.2000.078006667.x 11167229

[B61] Striph GG Hart WM Jr Olk RJ Modified grid laser photocoagulation for diabetic macular edema. The effect on the central visual field Ophthalmology 1988 95 12 1673 1679 10.1016/S0161-6420(88)32957-X 3231435

[B62] Boyer D Heier J Brown DM Clark WL Vitti R Berliner AJ Groetzbach G Zeitz O Sandbrink R Zhu X Vascular endothelial growth factor Trap-Eye for macular edema secondary to central retinal vein occlusion: six-month results of the phase 3 COPERNICUS study Ophthalmology 2012 119 5 1024 1032 10.1016/j.ophtha.2012.01.042 22440275

[B63] Brown DM Heier JS Clark WL Boyer DS Vitti R Berliner AJ Zeitz O Sandbrink R Zhu X Haller JA Intravitreal aflibercept injection for macular edema secondary to central retinal vein occlusion: 1-year results from the phase 3 COPERNICUS study Am J Ophthalmol 2013 155 3 429 437 e427 10.1016/j.ajo.2012.09.026 23218699

[B64] Holz FG Roider J Ogura Y Korobelnik JF Simader C Groetzbach G Vitti R Berliner AJ Hiemeyer F Beckmann K VEGF Trap-Eye for macular oedema secondary to central retinal vein occlusion: 6-month results of the phase III GALILEO study Br J Ophthalmol 2013 97 3 278 284 10.1136/bjophthalmol-2012-301504 23298885

[B65] Korobelnik JF Holz FG Roider J Ogura Y Simader C Schmidt-Erfurth U Lorenz K Honda M Vitti R Berliner AJ Intravitreal aflibercept injection for macular edema resulting from central retinal vein occlusion: one-year results of the phase 3 GALILEO study Ophthalmology 2013 121 1 202 208 24084497 10.1016/j.ophtha.2013.08.012

