# Supplementary material for: Efficacy and safety of widely used treatments for macular oedema secondary to retinal vein occlusion: a systematic review
Source: BMC Ophthalmol. 2014 Jan 21;14:7. doi: 10.1186/1471-2415-14-7 (PMC3904417; doi:10.1186/1471-2415-14-7)
Supplement: Additional file 1 — Database search strategy for MEDLINE and MEDLINE In-Process. [file 1471-2415-14-7-S1.docx]

**Appendix 1 Database search strategy for MEDLINE and MEDLINE In-Process**

1 macular edema/ (3044)

2 exp Edema/ (30716)

3 (macular adj3 (edema or oedema or odema)).ti,ab. (4733)

4 Retinal vein/ (1645)

5 Retinal Vein Occlusion/ (2322)

6 ((vein or veins or veinous) adj5 (occlusion$1 or occluded or obstruction$1 or obstructed or closed or closure$1 or stricture$1 or stenosis or stenosed or block or blocks or blockage$1 or blocking or embolism$1 or emboli) adj5 retina$1).ti,ab. (2560)

7 (crvo or cvo or rvo or brvo or bvo or crvome).ti,ab. (1071)

8 (branch vein adj5 occlu$).ti,ab. (222)

9 (central vein adj5 occlu$).ti,ab. (156)

10 or/1-9 (39433)

11 (bevacizumab or avastin or nsc 704865 or nsc704865).ti,ab,rn. (4072)

12 Antibodies, Monoclonal/ (153358)

13 antibodies/tu (1560)

14 vascular endothelial growth factors/ or vascular endothelial growth factor a/ (25799)

15 (vascular endothelial growth or vegf$ or antivegf$2).ti,ab. (33304)

16 (ranibizumab or lucentis or rhufab v2 or 347396-82-1).ti,ab,rn. (590)

17 Angiogenesis Inhibitors/ (10146)

18 Triamcinolone Acetonide/ (4369)

19 Triamcinolone acetonide.ti,ab,rn. (5023)

20 ivta.ti,ab,rn. (176)

21 exp Dexamethasone/ (40308)

22 ozurdex.ti,ab,rn. (3)

23 exp Light Coagulation/ (9974)

24 (photocoagulation or laser coagulation).ti,ab. (7400)

25 or/11-24 (250837)

26 animals/ not humans/ (3515697)

27 (editorial or letter or news).pt. (1096470)

28 10 and 25 (3069)

29 28 not (26 or 27) (2420)

30 limit 29 to English language (2001)

**Key:**

$ truncation symbol

/ subject heading

exp explode (subject heading)

tu. therapeutic use (subheading)

.ti. title

.ab. abstract

rn. CAS Registry/EC Number/Name

adj adjacent terms

adj5 terms within 5 words of each other

# or/1-9 combine sets 1 to 9 using OR
