# Supplementary material for: Efficacy and safety of widely used treatments for macular oedema secondary to retinal vein occlusion: a systematic review
Source: BMC Ophthalmol. 2014 Jan 21;14:7. doi: 10.1186/1471-2415-14-7 (PMC3904417; doi:10.1186/1471-2415-14-7)
Supplement: Additional file 2 — Assessment of study quality and risk of bias. [file 1471-2415-14-7-S2.docx]

**Appendix 2 – Assessment of study quality and risk of bias**

| **Study** | **Quality indicators** | | | | | | | |  |
| --- | --- | --- | --- | --- | --- | --- | --- | --- | --- |
|  | **Randomization** | **Allocation** | **Blinding** | **Groups similar at baseline** | **Loss to follow-up** | **Imbalance between groups** | **Intention-to-treat** | **Free of selective reporting** | **Score^a^ (from available data extraction)** |
| BRAVO [[32](#_ENREF_32)] | Unclear | Unclear | Double-blind | Yes | < 20% each group | No | Yes | Yes | Good (6/8) |
| CRUISE [[32](#_ENREF_32)] | Unclear | Unclear | Double-blind | Yes | < 20% each group | No | Yes | Yes | Good (6/8) |
| ROCC [[34](#_ENREF_34)] | Unclear | Unclear | Double-blind | Unclear | < 20% in total | No | No | Yes | Moderate (4/8) |
| GENEVA [[35](#_ENREF_35)] | Unclear | Adequate | Double-blind | Yes | < 20% all groups | No | Yes | Yes | Good (7/8) |
| Kupperman *et al.* [[36](#_ENREF_36)] | Unclear | Adequate | Double-blind | Yes | < 20% | No | Yes | Yes | Good (7/8) |
| BVOS [[6](#_ENREF_6)] | Adequate | Adequate | Single-blind | Yes | < 20% | No | Unclear | Yes | Good (7/8) |
| Battaglia *et al.* [[37](#_ENREF_37)] | Unclear | Unclear | At least single-blind | Yes | < 20% | Unclear | No | No | Poor (3/8) |
| CVOS [[10](#_ENREF_10)] | Unclear | Adequate | Single-blind | Yes | > 40% drop-out in both groups | No | NS | Yes | Moderate (5/8) |
| May *et al.* [[39](#_ENREF_39)] | Unclear | Unclear | Unclear | Yes | < 20% | No | NS | Yes | Moderate (5/8) |
| Laatikainen *et al.* [[38](#_ENREF_38)] | Adequate | Adequate | No | Unclear | < 20% | No | No | Yes | Moderate (5/8) |
| Russo *et al.* [[42](#_ENREF_42)] | Quasi-randomized | No | Unclear | Yes | None | No | Yes | Yes | Moderate (5/8) |
| Moradian *et al.* [[41](#_ENREF_41)] | Adequate | Adequate | Double-blind | Yes | None | No | Unclear | Yes | Good (7/8) |
| Faghihi *et al.* [[40](#_ENREF_40)] | Unclear | Unclear | Double-blind | Yes | Study not completed | Study not completed | Study not completed | Unclear | Poster only |

^a^Studies scored a point for every criterion for which their design was considered adequate.

BRAVO, Ranibizumab for the Treatment of Macular Edema after BRAnch retinal Vein Occlusion: Evaluation of Efficacy and Safety; CRUISE, Ranibizumab for the Treatment of Macular Edema after Central Retinal Vein OcclUsIon Study: Evaluation of Efficacy and Safety; ROCC, Study Comparing Ranibizumab to Sham in Patients with Macular Edema Secondary to Central Retinal vein OCClusion; GENEVA, Global EvaluatioN of implantable dExamethasone in retinal Vein occlusion with macular edemA; BVOS, Branch Retinal Vein Occlusion Study; CVOS, Central Retinal Vein Occlusion Study.
